# DIFFERENCES IN HEALTH AMONG LOW-INCOME OLDER IMMIGRANTS: DOES ETHNICALLY HOMOGENEOUS ENVIRONMENT MATTER?

**DOI:** 10.1093/geroni/igac059.1325

**Published:** 2022-12-20

**Authors:** BoRin Kim, ByeongJu Ryu, Jihye Baek, Sojung Park, Chung Hyeon Jeong, Jina Park

**Affiliations:** University of New Hampshire, Durham, New Hampshire, United States; Chuncheon Hyoja Social Welfare Center, Chuncheon-si, Kangwon-do, Republic of Korea; Washington University in St. Louis, St. Louis, Missouri, United States; Washington University in St. Louis, St. Louis, Missouri, United States; University of New Hampshire, Durham, New Hampshire, United States; Washington University in St. Louis, St. Louis, Missouri, United States

## Abstract

Older immigrants comprised 14% of the US older population, which is predicted to continue to rise. Despite the importance of ethnic attachment in older immigrants’ well-being, little is known how ethnically homogeneous housing environment affect older adults’ health. This study aims to fill the gaps in knowledge on low-income older immigrants living in subsidized senior housing and to explore the associations between immigrant status, senior housing environments, and self-rated health among this population. Our survey data was collected from 18 subsidized senior housing communities in IL, MO, and NH (N=459). Bilingual surveyors fluent in Chinese, Russian, and Korean were also available for the participants with limited English proficiency as the sampled housings were the multi-ethnicity properties. Hierarchical Linear Modeling was employed for our analysis to address our participants were clustered within senior housing.More than a half (56%) of our sample lived in ethnically homogeneous senior housing (either all immigrants or all non-immigrants). Immigrants consist of 53% of our sample, and 48% of them lived in ethnically homogeneous housing. Our finding showed that immigrants were more likely to report low level of self-rated health (OR=0.885, p< 0.01). However, ethnically homogeneous senior housing environments positively influence the association between immigrant status and self-rated health (OR=1.219, p<.05).This study highlights the importance of ethnically homogeneous living environment for low-income older immigrants in senior housing. Although ethnic diversity may be beneficial in broader society, concentration of ethnic minority older immigrants could play an important role in terms of social support and service delivery.

